# Analysis of human B-cell responses following ChAd63-MVA MSP1 and AMA1 immunization and controlled malaria infection

**DOI:** 10.1111/imm.12226

**Published:** 2014-03-11

**Authors:** Sean C Elias, Prateek Choudhary, Simone C de Cassan, Sumi Biswas, Katharine A Collins, Fenella D Halstead, Carly M Bliss, Katie J Ewer, Susanne H Hodgson, Christopher J A Duncan, Adrian V S Hill, Simon J Draper

**Affiliations:** 1 The Jenner Institute, University of OxfordOxford, UK; 2 Centre for Clinical Vaccinology and Tropical Medicine, The Jenner Institute, University of Oxford, Churchill HospitalOxford, UK

**Keywords:** antibodies, B cell, memory, parasitic protozoan, vaccination

## Abstract

Acquisition of non-sterilizing natural immunity to *Plasmodium falciparum* malaria has been shown in low transmission areas following multiple exposures. However, conflicting data from endemic areas suggest that the parasite may interfere with the induction of effective B-cell responses. To date, the impact of blood-stage parasite exposure on antigen-specific B cells has not been reported following controlled human malaria infection (CHMI). Here we analysed human B-cell responses in a series of Phase I/IIa clinical trials, which include CHMI, using candidate virus-vectored vaccines encoding two blood-stage antigens: merozoite surface protein 1 (MSP1) and apical membrane antigen 1 (AMA1). Previously vaccinated volunteers show boosting of pre-existing antigen-specific memory B-cell (mBC) responses following CHMI. In contrast, unvaccinated malaria-naive control volunteers developed an mBC response against MSP1 but not AMA1. Serum IgG correlated with the mBC response after booster vaccination but this relationship was less well maintained following CHMI. A significant reduction in peripheral MSP1-specific mBC was observed at the point of diagnosis of blood-stage infection. This was coincident with a reduction in peripheral blood B-cell subsets expressing CXCR3 and elevated serum levels of interferon-*γ* and CXCL9, suggesting migration away from the periphery. These CHMI data confirm that mBC and antibody responses can be induced and boosted by blood-stage parasite exposure, in support of epidemiological studies on low-level parasite exposure.

## Introduction

It has been widely reported that naturally acquired immunity (NAI) to human *Plasmodium falciparum* malaria is non-sterile and slow to acquire, requiring repeated infections over a number of transmission seasons. More recent studies have further refined this view, suggesting differences in immune repertoires acquired by individuals living in highly endemic areas compared with those where malaria infection is less frequent.^1^ These differences in acquisition of NAI not only make it more complex to elucidate immune mechanisms of protection, but make designing a broadly protective vaccine for *P. falciparum* malaria based on these mechanisms all the more challenging. It also remains possible that protection mediated through subunit vaccination will be achieved by mechanisms that are not strongly associated with NAI.^2^ In either case, it remains important for clinical vaccine developers to understand how natural malaria exposure could modulate vaccine-induced immune responses; however, to date, little information exists to address this question.

The blood-stage malaria antigens merozoite surface protein 1 (MSP1) and apical membrane antigen 1 (AMA1) are exposed as the merozoite invades target erythrocytes, and are considered important blood-stage vaccine candidates, especially owing to their association with protective immunity in pre-clinical studies of mice^3^,^4^ and non-human primates.^5^–^7^ Protection is primarily associated with the induction of high-titre antibodies and, to a lesser extent, T-cell activity. These antigens have also been shown to induce antibody and memory B cell (mBC) responses following natural parasite exposure in mice^8^,^9^ and humans,^10^–^12^ with both expanding gradually upon repeated exposures. In areas where *P. falciparum* malaria transmission is low, antibody and mBC responses have been shown to be induced and long-lived^13^,^14^ with the breadth (but not magnitude) of the mBC response expanding with age and exposure.^15^ In other studies antibody and mBC responses to these antigens have been reported to be short-lived or below detection in the peripheral blood.^16^,^17^ It has also been observed that malaria infection in Kenyan children can lead to disturbances in peripheral B-cell homeostasis;^18^,^19^ whereas continued exposure can lead to expansion of a so-called ‘atypical mBC’ subset with reduced proliferative capacity.^20^,^21^ Further evidence for the effect of parasite infection on the mBC compartment comes from murine studies where it has been proposed that long-term protection provided by vaccine-induced mBC and long-lived plasma cells (LLPC) specific for MSP1 can be ablated by *Plasmodium yoelii* infection.^22^ These data, in conjunction with the known gradual acquisition of NAI, have led many to propose that malaria infection induces dysregulation of B-cell function.^23^

Studying the mechanisms by which antibody responses and the mBC compartment are acquired and maintained is naturally more difficult in human studies than in mice. Due to ethical considerations and the invasiveness of required procedures, access to human lymph nodes, spleen and bone marrow is rarely possible so the focus of most studies is on peripheral blood. The most widely used assay for investigating peripheral mBC responses is the mBC ELISPOT.^24^ This assay allows identification of antigen-specific mBC-derived plasma cells by *ex vivo* ELISPOT assay following a 6-day polyclonal culture, and has been used to measure both naturally acquired^10^,^12^,^14^,^15^ and vaccine-induced responses.^25^ This method was used here to investigate the induction of mBC responses following vaccination of healthy UK adults with recombinant chimpanzee adenovirus 63 (ChAd63) and modified vaccinia virus Ankara (MVA) vectors encoding the blood-stage *P. falciparum* malaria antigens MSP1^26^ or AMA1.^27^ We have previously reported that this regimen induces strong antibody and T-cell responses for both antigens as well as exhibiting a good safety profile in Phase Ia clinical trials.^28^,^29^ A Phase IIa trial, assessing the efficacy of these vaccines following a controlled human malaria infection (CHMI) has also been completed.^30^ These clinical trials provided a unique opportunity to assess the impact of blood-stage parasite exposure on vaccine-induced as well as bystander mBC populations in a controlled human infection setting. In contrast, all other reported studies to-date have assessed human B-cell immunomodulation in the context of natural malaria exposure in the field, where differences in numbers of infectious episodes and circulating strains can make data interpretation more difficult.

Here we report that vaccination of healthy adult volunteers with ChAd63 and MVA vectors encoding the blood-stage *P. falciparum* malaria antigens MSP1 and AMA1 induces mBC responses comparable to similar vaccine studies using protein-in-adjuvant vaccines.^25^ Following CHMI, an mBC response to the 19 kDa C-terminus of MSP1 (MSP1_19_) develops in malaria-naive infectivity control volunteers, whereas responses to both the MSP1_19_ and AMA1 antigens are boosted in previously vaccinated individuals. Interestingly, mBC responses against MSP1_19_ remained significantly elevated for the duration of the study, following on from a notable decrease of peripheral MSP1_19_-specific mBC at the time of peak blood-stage parasitaemia (coincident with malaria diagnosis and treatment), whereas bystander responses to an irrelevant antigen (diphtheria toxoid) remained unaffected. Phenotyping by flow cytometry showed that B-cell subsets are relatively stable during blood-stage infection, but that at the time of peak infection (day of diagnosis; DoD) there is a distinct reduction in subsets expressing the chemokine receptor CXCR3, most notably in the classical and activated mBC populations. This was evident only in vaccinated individuals and not in unvaccinated controls, and associated with elevated levels of serum interferon-*γ* (IFN-*γ*) and the CXCR3 ligand, monokine-induced by IFN-*γ* (MIG/CXCL9). These first data from the CHMI model therefore suggest that *P. falciparum* infection does not ablate malaria vaccine-induced or bystander B-cell memory, and can instead contributes to its maintenance through recruitment and activation of vaccine-induced antigen-experienced mBC at sites of infection.

## Materials and methods

### Immunization groups and peripheral blood mononuclear cells

Frozen peripheral blood mononuclear cell (PBMC) samples were used throughout this study and were obtained from Phase Ia safety and immunogenicity clinical trials for the MSP1^28^ and AMA1^29^ candidate vaccines, as well as three Phase IIa efficacy studies (called MAL034A, MAL034B and VAC039) where immunized volunteers underwent CHMI with vaccine-homologous *P. falciparum* 3D7 clone sporozoites delivered by mosquito bite (see Supporting information, Fig. S1).^30^ In all cases, the two antigens were separately delivered by a heterologous prime-boost immunization regimen consisting of a priming intramuscular (i.m.) vaccination with a recombinant replication-deficient ChAd63 vector (doses: 5 × 10^9^ to 5 × 10^10^ viral particles), followed 8 weeks later by an intramuscular boosting vaccination with the MVA vector (doses: 1·25 × 10^8^ to 5 × 10^8^ plaque forming units) recombinant for the same antigen. All necessary regulatory and ethical approvals were granted as previously described,^28^–^30^ and the trials were registered with ClinicalTrials.gov. All volunteers gave written informed consent before participation, and the studies were conducted according to the principles of the Declaration of Helsinki and in accordance with Good Clinical Practice. All volunteers participating in these clinical trials gave permission for samples to be used for exploratory immunology analysis. The PBMC samples from the trials were all prepared and frozen as previously described.^28^

### Vaccine antigens and proteins

The composition of the vaccine inserts for MSP1^26^ and AMA1^27^,^32^ used in both the ChAd63 and MVA vaccine vectors have been previously described. In the case of AMA1, a bivalent transgene was optimized to consist of the 3D7 and FVO strain alleles fused in tandem; whereas for MSP1 an insert was designed comprising both the 3D7/MAD20 and Wellcome alleles of the dimorphic 42 kDa C-terminal region (MSP1_42_/sequence blocks 16 + 17) fused in tandem and preceded by the naturally conserved regions of MSP1 sequence (blocks 1, 3, 5 and 12).^33^ The MSP1_42_ region is composed of an N-terminal 33 kDa region (MSP1_33_, block 16) followed by a C-terminal 19 kDa region (MSP1_19_, block 17).

The recombinant MSP1_19_ proteins used in the ELISPOT assays in this study were produced in *Escherichia coli* and have been previously reported.^28^ The proteins consist of the MSP1_19_ region fused to glutathione *S*-transferase. Proteins representing both alleles of MSP1_19_ used in the vaccine insert, ETSR (3D7) and QKNG (Wellcome), were used in a 50 : 50 ratio (final concentration 5 μg/ml each) in the mBC and antibody-secreting cell (ASC) ELISPOT assays. The recombinant AMA1 FVO protein used in this study has been previously reported,^29^ and the 3D7 AMA1 protein^34^ was a kind gift from Dr Y. Wu (National Institute of Allergy and Infectious Diseases, National Institutes of Health, Bethesda, MD). Proteins representing both alleles used in the vaccine insert (3D7 and FVO) were used in a 50 : 50 ratio (final concentration 5 μg/ml each) in the mBC and ASC ELISPOT assays.

### ELISA

Standardized ELISAs were performed for both antigens as previously reported.^28^,^29^ Total serum IgG antibodies were measured against the proteins representing the 3D7 alleles of MSP1_19_ and AMA1 (homologous to the CHMI strain).

Interferon-*γ* ELISA was performed using ‘Human IFN gamma ELISA Ready-SET-Go!’ (eBioscience, Hatfield, UK) as per the manufacturer's instructions. At most time-points neat serum or a 1 : 5 dilution was tested, but at DoD up to 1 : 100 serum dilution was required. CXCL9 (MIG) ELISA was performed using ‘Human MIG Instant ELISA’ (eBioscience) as per the manufacturer's instructions. For the time-point one day prior to challenge (dC-1) neat serum was used, whereas for DoD up to 1 : 5 serum dilution was required.

### Memory B-cell ELISPOT

The mBC ELISPOT protocol was based on a widely published method.^24^ Frozen PBMC were thawed, counted and resuspended to 2 × 10^6^/ml. Five hundred microlitres of cells were added to each well of a 24-well plate. These cells were cultured with 500 μl of a polyclonal B-cell stimulation mix containing *Staphylococcus aureus* Cowans strain Pansorbin cell (1/2400; Calbiochem, Merck, Darmstadt, Germany), the human Toll-like receptor agonist CpG ODN-2006 (5 μg/ml; Invivogen, San Diego, CA) and pokeweed mitogen (1/6000; Sigma-Aldrich Company Ltd, Dorset, UK) for 6 days, allowing mBC to differentiate into ASC. One well per time-point was also cultured in the absence of stimulation mix as a control. On day 5 of the experiment, ELISPOT plates were coated with MSP1_19_ or AMA1 protein to measure the antigen-specific response and polyvalent goat-anti human IgG (Caltag, Buckingham, UK) to measure the total IgG response. A separate plate was coated with a non-malaria vaccine protein/antigen [diphtheria toxoid (DT), National Institute for Biological Standards and Control]. PBS-coated wells were used as a negative control. The mBC were also tested against recombinant glutathione *S*-transferase (the tag fused to MSP1_19_ protein), for which all responses were negative (data not shown). On day 6, cultured cells were harvested and transferred to the ELISPOT plate at 2 × 10^5^ cells per well, with additional dilutions of 1 : 2 for the antigen-specific response wells and 1 : 100 and 1 : 1000 for the total IgG response wells. After overnight culture (18–20 hr) cells were discarded, the plate was washed and an anti-human IgG (*γ*-chain) antibody was conjugated to alkaline phosphatase (Calbiochem) added for 4 hr. The plates were then washed again and developed using development buffer (Europa Bioproducts). The substrate was left to develop for 3–5 min until spots were clearly visible, at which point the plate was washed in water and left to dry. Plates were counted using an AID ELISPOT plate reader.

### Antibody-secreting cell ELISPOT

ELISPOT plates were coated with MSP1_19_ or AMA1 protein to measure the antigen-specific response. PBS-coated wells were used as a negative control and DT-coated wells were used as a non-malaria antigen control. PBMC were freshly prepared as previously described,^28^ and plated at 2·5 × 10^5^ cells per well with additional dilutions of 1 : 2 for the antigen-specific response. After overnight culture (18–20 hr) cells were discarded, and the plate was developed and counted by the same method as mBC ELISPOT.

### Flow cytometry

Phenotypic analysis was performed on frozen PBMC from six MSP1-vaccinated volunteers in the VAC039 clinical trial^30^ at time-points d56, d63, dC−1, dC+7, DoD and dC+35 (see Supporting information, Fig. S1). Three out of six vaccinated volunteers were also measured at dC+11. All vaccinated volunteers received an MSP1 vaccination regimen before CHMI. Five CHMI unvaccinated control volunteers from the VAC039 clinical trial were also analysed at the dC−1, dC+7, DoD and dC+35 time-points for direct comparison. Analyses were performed using mouse anti-human fluorophore-conjugated monoclonal antibodies specific for B-cell and migratory markers: CD10 allophycocyanin (HI10a), CD19 phycoerythrin-Cy7 (SJ25C1), IgG phycoerythrin (G18-145), CXCR3 Alexa-Fluor 488 (1C6) (BD Biosciences, Franklin Lakes, NJ); CD27 Qdot 605 (CLB-27/1) (Invitrogen, Eugene, OR); CD20 allophycocyanin (2H7), CD21 efluor450 (HB5), CD38 phycoerythrin-Cy5 (HIT2) (eBioscience). This eight-colour panel was used to identify B-cell subsets as follows: immature B cells (CD19^+^, CD10^+^), naive B cells (CD19^+^, CD10^−^, CD21^+^, CD27^−^); classical mBC (CD19^+^, CD10^−^, CD21^+^, CD27^+^); atypical mBC (CD19^+^, CD10^−^, CD21^−^, CD27^−^); activated mBC (CD19^+^, CD10^−^, CD21^−^, CD27^+^, CD20^+^, CD38^−^); and plasmablast/ASC (CD19^+^, CD10^−^, CD21^−^, CD27^+^, CD20^−^, CD38^+^; see Supporting information, Fig. S2). IgG^+^ staining was also quantified for classical and atypical mBC subsets. CXCR3^+^ staining was quantified for classical, atypical and activated mBC subsets as well as naive B cells. In Fig. S3 (see Supporting information), a small number of events from the following subsets were excluded from the analysis because of undefined phenotypes, (CD19^+^, CD10^−^, CD21^−^, CD27^+^, CD20^−^, CD38^−^; and CD19^+^, CD10^−^, CD21^−^, CD27^+^, CD20^+^, CD38^+^). Analysis was performed on an LSRII Flow Cytometer (BD Biosciences). Gating strategy for fluorochromes was determined using F-1 analysis and unstained controls.

### Statistics

Data were analysed using GraphPad Prism version 5.03 for Windows (GraphPad Software Inc., San Diego, CA). Wilcoxon matched-pairs signed rank tests were carried out to compare volunteer responses (paired responses) between selected time-points. Alternatively Friedman tests were used when comparing multiple time-points from the same group (paired responses). Mann–Whitney *U*-tests were used to compare between two different volunteer groups at comparable time-points. Spearman's rank was used to analyse correlations. Statistical significance was considered at *P *≤* *0·05.

## Results

### ChAd63-MVA prime-boost vaccination induces antigen-specific mBC responses

**Figure 1:**
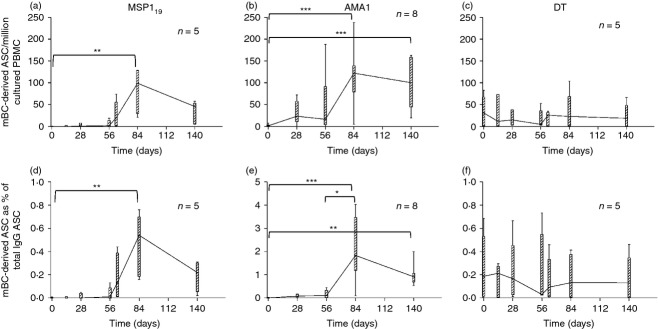
Memory B-cell (mBC) responses over time in volunteers receiving ChAd63-MVA merozoite surface protein 1 (MSP1) or apical membrane antigen 1 (AMA1) prime-boost vaccination. Box and whisker plots show the median, range and interquartile range of antigen-specific mBC-derived ASC responses measured over time using the mBC ELISPOT assay. Responses are defined as mBC-derived antigen-secreting cells (ASC) per million cultured peripheral blood mononuclear cells (PBMC) specific for (a) MSP1_19_, (b) AMA1 and (c) diphtheria toxoid (DT); or as mBC-derived ASC as a % of total IgG^+^ ASC for the same antigens (d–f). Assays were performed at the indicated time-points from frozen PBMC; MSP1 vaccinees (*n *=* *5), AMA1 vaccinees (*n *=* *8); DT responses are shown from MSP1-vaccinated volunteers (*n *=* *5). Assays could not be performed at the d14 and d63 time-points for AMA1-vaccinated volunteers because of the limited PBMC sample availability. Friedman Test, with Dunn's post-test, *P <* *0·05, **P <* *0·01 and ***P <* *0·001.

We have previously reported T-cell and antibody immunogenicity in a series of three Phase I/IIa clinical trials using the ChAd63-MVA vectors encoding MSP1 or AMA1^28^–^30^ (trial outlines shown in Fig. S1). Volunteers were primed by recombinant ChAd63 immunization on day 0 (d0) and boosted 56 days later with the MVA vector encoding the same antigen. Here we report a detailed assessment of vaccine-induced B-cell responses. Initially mBC ELISPOT assays were performed at each time-point (Fig. S1a) for MSP1-vaccinated volunteers,^28^ to identify the kinetics of the response. After ChAd63 priming (d28/56) the IgG^+^ mBC response was not significantly different from that on d0 (Fig. 1a). However, following the MVA boost there was an increase in peripheral blood mBC peaking at d84 (28 days post-boost), with a median response of 99 mBC-derived ASC per million cultured PBMC which was significantly different from d0. This response subsequently contracted but was maintained at a resting median level of 46 at d140. It is also common to report antigen-specific IgG^+^ mBC-derived ASC as a % of total IgG^+^ mBC-derived ASC. In this case the d84 median was 0·54% (Fig. 1d), again significantly higher than d0.

In the case of AMA1,^29^ ChAd63 priming induced a more detectable response at d28 that was maintained up to the day of boosting (d56). Following the MVA boost there was a significant increase (in comparison to d0) up to a median of 122 mBC-derived ASC per million cultured PBMC at d84 (Fig. 1b), before the response contracted to a resting level of 101 at d140, which maintained significance over baseline. When calculating IgG^+^ mBC-derived ASC as a % of total IgG^+^ mBC-derived ASC, a similar kinetic was observed in addition to a significant increase from pre-boost levels (d56) to post-boost peak (d84; Fig. 1e).

The resting mBC response was also measured to DT as a non-malaria antigen control. No bystander activation of this population was observed following ChAd63-MVA vaccination and the median response was stable at approximately 25 mBC-derived ASC per million cultured PBMC (Fig. 1c), or 0·18% of IgG^+^ mBC-derived ASC (Fig. 1f). These data demonstrated the induction of antigen-specific mBC responses by virus-vectored vaccination, with no apparent effect on pre-existing bystander responses.

### Controlled *P. falciparum* infection induces mBC to MSP1_19_ but not AMA1 in unvaccinated infectivity control volunteers

**Figure 2:**
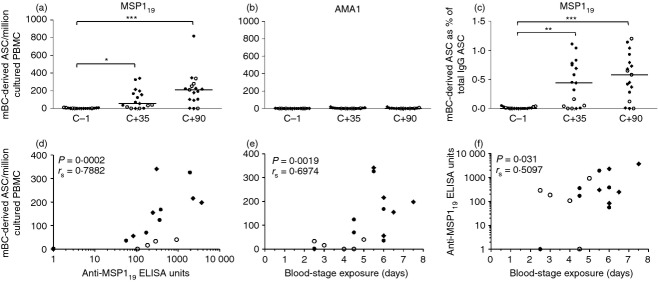
Memory B-cell (mBC) responses in control unvaccinated volunteers receiving controlled human malaria infection (CHMI). Antigen-specific mBC-derived antigen-secreting cells (ASC) per million cultured peripheral blood mononuclear cells (PBMC) were quantified for the (a) 19 kDa C-terminus of merozoite surface protein 1 (MSP1_19_) and (b) apical membrane antigen 1 (AMA1 antigens in malaria-naive unvaccinated control volunteers before (dC−1) and following a single CHMI (dC+35 and dC+90). The mBC-derived ASC as a % of total IgG^+^ ASC for MSP1_19_ are also shown (c). Data are presented as dot plots showing individual and median responses. Time-points were compared using Friedman Test, with Dunn's post-test, *P <* *0·05, **P <* *0·01, and ***P <* *0·001. (d) Correlation at dC+35 between MSP1_19_-specific mBC-derived ASC per million cultured PBMC and anti-MSP1_19_ ELISA units; (e) MSP1_19_-specific mBC-derived ASC per million cultured PBMC and duration of blood-stage parasite exposure; and (f) anti-MSP1_19_ ELISA units and duration of blood-stage parasite exposure. Spearman rank correlation coefficient and *P*-values are shown (*n *=* *17). Blood-stage exposure was calculated as the number of days from dC+6·5, the predicted time-point of first blood-stage parasitaemia, to time-point of diagnosis. Data are presented and pooled from three separate CHMI trials: MAL034A (closed diamonds, *n *=* *6), MAL034B (closed circles, *n *=* *6) and VAC039 (open circles, *n *=* *5).

In three Phase IIa challenge trials, volunteers were vaccinated and then exposed to a controlled *P. falciparum* infection to test vaccine efficacy^30^,^31^ (Fig. S1b).^30,31^ Infectivity control groups (*n *=* *6 malaria-naive individuals per study, *n *=* *17 assayed here in total) included volunteers who were exposed to controlled *P. falciparum* infection without previous vaccination (Fig. S1c). According to the trial protocols, volunteers were exposed to five infectious mosquito bites and then followed up to a maximum of 21 days with daily blood-films and quantitative PCR to measure blood parasitaemia. *Plasmodium falciparum* parasites emerge from the liver at around dC+6·5–7 and commence the blood-stage of infection, which is responsible for subsequent clinical symptoms.^35^ All control volunteers were diagnosed and treated with anti-malarial drugs as per protocol, with a median time to microscope-patent diagnosis of 11 days post-CHMI (range 9–14 days^30^,^31^). Antigen-specific mBC ELISPOT assays for both MSP1_19_ and AMA1 were subsequently performed on frozen PBMC at the dC−1 time-point (1 day before CHMI), dC+35 and dC+90 following CHMI. Fourteen of 17 volunteers showed a positive IgG^+^ mBC response to MSP1_19_ at dC+90 (Fig. 2a), but no volunteers showed a positive mBC response to AMA1 (Fig. 2b). Responses against MSP1_19_ at dC+35 and dC+90 were significantly higher than the dC−1 baseline for both mBC-derived ASC per million cultured PBMC (Fig. 2a) and as a % IgG^+^ mBC-derived ASC (Fig. 2c). Interestingly, the MSP1_19_-specific responses induced by primary parasite exposure were comparable in magnitude (if not stronger) to those induced by ChAd63-MVA MSP1 vaccination (Fig. 1a,d). When serum total IgG responses to these two antigens were measured, the three volunteers with no detectable mBC responses to MSP1_19_ also showed no detectable antibody to MSP1_19_, whereas only a minority of volunteers showed serum antibodies to AMA1 that were marginal and just above the limit of detection (Biswas S, Choudhary P, Draper SJ, in preparation). For the volunteers who did mount *de novo* anti-MSP1_19_ antibody responses, there was a strong correlation between MSP1_19_ serum total IgG and the mBC response at dC+35 (Fig. 2d, *r*_s_ = 0·79, *P *=* *0·0002), which was maintained at dC+90 (*P *=* *0·0047, *r*_s_ = 0·80; data not shown). There was also a significant correlation between the mBC response (Fig. 2e) and serum antibody (Fig. 2f) and the number of days volunteers were exposed to blood-stage parasitaemia. This was calculated as number of days from dC+6·5 (earliest expected time when parasites leave the liver) to the time-point of blood-film diagnosis (DoD). These data suggest that in the context of natural blood-stage infection, antibody and mBC responses against MSP1_19_ dominate over those against AMA1, and at least during an acute primary infection, the magnitude of the response corresponds to the duration of infection.

### Controlled *P. falciparum* infection boosts the vaccine-induced mBC response

**Figure 3:**
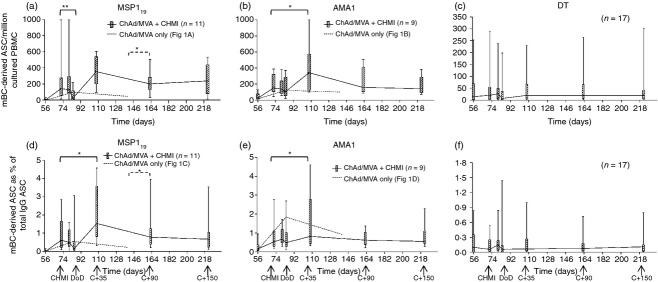
Memory B-cell (mBC) responses in vaccinated volunteers receiving controlled human malaria infection (CHMI). Box and whisker plots show the median, range and interquartile range of antigen-specific mBC-derived antigen-secreting cell (ASC) responses measured over time using the mBC ELISPOT assay. Responses are defined as mBC derived ASC per million cultured peripheral blood mononuclear cells (PBMC) specific for (a) 19 kDa C-terminus of merozoite surface protein 1 (MSP1_19_), (b) apical membrane antigen 1 (AMA1) and (c) diphtheria toxid (DT); or as mBC-derived ASC as a % of total IgG^+^ ASC for the same antigens (d–f). Assays were performed at the indicated time-points from frozen PBMC; MSP1 vaccinees (*n *=* *11) and AMA1 vaccinees (*n *=* *9) are shown by solid lines; DT responses are shown from vaccinated volunteers in both the MSP1 and AMA1 groups (*n *=* *17). Day of diagnosis (DoD) is plotted at d84 as the average diagnosis time-point. Responses from vaccinated volunteers who did not receive CHMI are shown in (a, b, d, e) from the previous Phase Ia trials (median response from Fig. 1a, b, d, e dashed line). Differences between time-points in the Phase IIa trial were analysed by Wilcoxon matched-pairs signed rank test (solid lines). Differences between resting levels in the Phase IIa and Phase Ia trials were analysed by Mann–Whitney *U*-test (dashed lines), *P <* *0·05 and **P <* *0·01. Differences between all time-points for DT performed using Friedman test with Dunn's post-test.

In two of the Phase IIa studies, volunteers were also vaccinated with the MSP1 or AMA1 virus-vectored vaccines and then exposed to CHMI (Fig. S1b). Although no protection was observed, a subset of volunteers displayed a delay in time to diagnosis.^30^ Given the conflicting evidence from immuno-epidemiological studies and rodent malaria models about the possible effect(s) of parasite exposure on B cells,^23^ we sought to assess the effect of CHMI on vaccine-induced and bystander mBC populations. Volunteers vaccinated with ChAd63-MVA MSP1 showed a median response before CHMI (dC−1/d72) of 142 mBC-derived ASC per million cultured PBMC (Fig. 3a). With the exception of one high responding individual, responses were similar to previous median results seen at d84 in the Phase Ia trial (Fig. 3a, dashed line). Following CHMI, responses remained level during the liver-stage of infection up to dC+7/d80; however, at DoD (plotted at d84) a significant decrease (*P *=* *0·0059) in peripheral mBC was observed (down to a median level of 22). Following drug treatment and resolution of infection, the peripheral mBC compartment recovered and showed a boosting of the response (median of 353 measured at dC+35/d108). This boosting did not reach significance for the measure of mBC-derived ASC per million PBMC (due to the one high responder at dC−1), but was significant when comparing antigen-specific cells as a % of total IgG^+^ (0·61% versus 1·54%, *P *=* *0·016; Fig. 3d). In this trial volunteers were followed up to 150 days post-CHMI (d223), providing an opportunity to assess the maintenance of the mBC population. In this group of vaccinated and infected volunteers, the resting level of mBC was 200 at dC+90/d163 and this was maintained at 223 at dC+150/d223, indicating overall that parasite exposure had boosted the MSP1_19_-specific mBC compartment and that this response was maintained for the duration of the study. When compared with vaccinated only volunteers (without CHMI), there was a significant difference (*P *=* *0·011) in resting level responses between d140 in the Phase Ia trial and dC+90/d163 (the closest equivalent time-point post-CHMI in the Phase IIa trial). The same result was observed for the antigen-specific mBC response as a % of total IgG^+^ mBC (*P *=* *0·013; Fig. 3d).

In a second group, volunteers were vaccinated with ChAd63-MVA AMA1 before CHMI. In this case, the kinetic of the mBC response differed in comparison to volunteers vaccinated with MSP1 (Fig. 3b). The median response before CHMI at dC−1 was 153, comparable to both MSP1-vaccinated volunteers and the previous AMA1-vaccinated volunteers in the Phase Ia trial (Fig. 3b, dashed line). However, unlike for MSP1-vaccinated volunteers, there was no significant reduction of AMA1-specific peripheral blood mBC at the time of diagnosis, with the median response only dropping slightly to 106. Following drug treatment and resolution of infection the mBC compartment also expanded significantly in AMA1-vaccinated volunteers (*P *=* *0·02) to a peak of 342 at dC+35 in comparison to dC−1, a magnitude that was highly comparable to that observed in MSP1 vaccinees. This was also significant for mBC measured as a % of total IgG^+^ mBC (*P *=* *0·0078; Fig. 3e). Subsequently, the median resting level of AMA1-specific mBC was 158 at dC+90 and 145 at dC+150. However, unlike for MSP1, this level was not significantly different from the resting level following vaccination only in the Phase Ia trial when comparing d140 and dC+90/d163. The same observation was made with respect to antigen-specific mBC as a % of total IgG^+^ mBC (Fig. 3e).

Bystander mBC responses were also measured over time against a non-malaria antigen (DT) in 17/20 vaccinees. Although there was a trend for lower peripheral DT-specific mBC-derived ASC at the time of malaria diagnosis (Fig. 3c) this was not significantly different from the responses at all other time-points. The same result was observed for DT-specific mBC-derived ASC as a % of total IgG^+^ (Fig. 3f). Otherwise responses were stable over time, at median levels comparable to that previously seen (Fig. 1c,f). CHMI therefore appears to have minimal, if any, impact on bystander mBC populations but could boost existing malaria-specific vaccine-induced mBC responses.

### MSP1 and AMA1 vaccine co-administration induces mBC responses to both antigens

**Figure 4:**
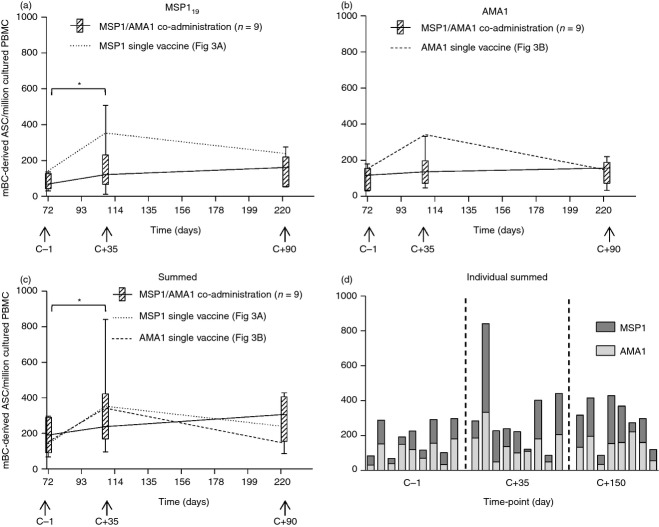
Memory B-cell (mBC) responses in volunteers receiving both vaccines and controlled human malaria infection (CHMI). Box and whisker plots show the median, range and interquartile range of antigen-specific mBC-derived antigen-secreting cell (ASC) responses measured over time using the mBC ELISPOT assay, in volunteers co-administered with the merozoite surface protein 1 (MSP1) and apical membrane antigen 1 (AMA1) vaccines (*n *=* *9), at the key time-points dC−1 (d72), dC+35 (d108) and dC+90 (d223). Responses are defined as mBC-derived ASC per million cultured peripheral blood mononuclear cells (PBMC) specific for (a) 19 kDa C-terminus of MSP1 (MSP119), (b) AMA1, and (c) MSP119 + AMA1 summed. The dotted line in (a) and (c) represents the median response from MSP1-only vaccinated volunteers (Fig. 3a). The dashed line in (b) and (c) represents the median response from AMA1-only vaccinated volunteers (Fig. 3b). Responses were compared with those in volunteers receiving single antigen vaccination and CHMI by Mann–Whitney *U*-test. (d) Individual breakdown of summed responses for each volunteer in the vaccine co-administration group at the three time-points. Differences between time-points in the Phase IIa trial were analysed by Wilcoxon matched-pairs signed rank test, *P <* *0·05 and **P <* *0·01.

A third group of volunteers in the Phase IIa trial received both the MSP1 and AMA1 virus-vectored vaccines which were co-administered in opposite arms (but at equal doses to the first two groups of volunteers meaning that overall the vector dose was double). We have previously reported that these volunteers showed a significant reduction in both *ex vivo* T-cell responses and ELISA antibody titres to both antigens following the MVA boost vaccination, in comparison to the single vaccine administration groups, but that responses to MSP1 appeared immuno-dominant to those against AMA1.^30^ Given that the infectivity control volunteers showed *de novo* induction of MSP1_19_-specific, but not AMA1-specific, mBC responses following the context of natural parasite exposure, it was of interest to also assess the effect of vaccine antigen co-administration on the induced mBC responses. The numbers of mBC-derived ASC were quantified by ELISPOT assay for these volunteers at dC−1/d72 (peak post-boost), dC+35/d108 (peak post-CHMI) and dC+150/d223 (resting level) for both MSP1_19_ (Fig. 4a) and AMA1 (Fig. 4b). Responses were significantly boosted by CHMI at dC+35 compared with dC−1 for MSP1 (*P *=* *0·039) but not for AMA1. Similar to the results for T-cell and serum IgG responses,^30^ individual antigen-specific mBC responses in this group tended to be lower at dC+35 (MSP1: 121, AMA1: 136) compared with responses in the equivalent individual vaccine groups (MSP1: 353, AMA1: 342), but despite disparity in medians, this did not reach significance (*P *=* *0·224 and 0·077, respectively). No significant differences were observed at the dC−1 or dC+150 time-points, although here the medians were more comparable. When antigen-specific responses were summed in the co-administration vaccine group, the total vaccine-induced response was more comparable to the responses induced in the single vaccination groups at all time-points (Fig. 4c), suggesting that mBC responses were susceptible to immune interference following vaccine co-administration. However, unlike following natural parasite exposure in malaria-naive individuals, mBC responses could be induced by vaccination against both antigens (Fig. 4d), although natural exposure only appeared to increase the MSP1_19_-specific population in the long term, as observed with single antigen vaccination. The mBC responses were also measured against the non-malaria antigen (DT) in the vaccine co-administration group, with results again confirming no impact of CHMI (data not shown).

### MSP1_19_-specific mBC responses correlate with antibodies post-MVA vaccination but not following CHMI

**Figure 5:**
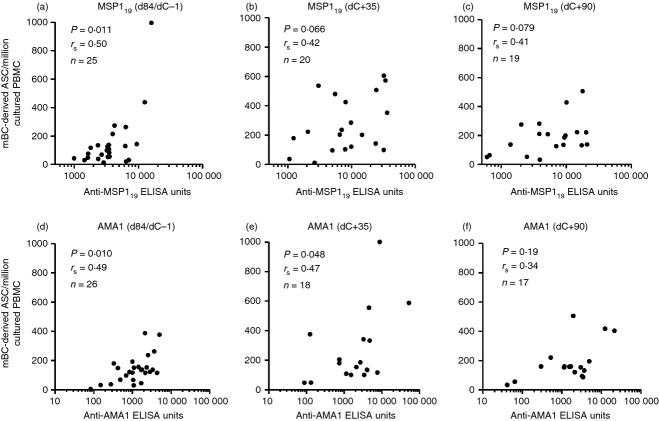
Relationship between peripheral memory B-cell (mBC) responses and serum IgG in vaccinees before and after controlled human malaria infection (CHMI). Correlation of the 19 kDa C-terminus of merozoite surface protein 1 (MSP1_19_)-specific mBC-derived antigen-secreting cell (ASC) per million cultured peripheral blood mononuclear cells (PBMC) and levels of serum IgG reported as anti-MSP1_19_ ELISA units. Results are shown (a) post ChAd63-MVA vaccination (dC−1/d84); (b) dC+35 post-CHMI; and (c) dC+90 post-CHMI. (d–f) show the same results for AMA1. Spearman rank correlation coefficient, *P* and *n* values are shown for all analyses.

Having observed that serum IgG responses correlated with mBC responses in unvaccinated infectivity controls post-CHMI (Fig. 2d), we also sought to investigate this relationship in vaccinees. Given that short-lived plasma cells (SLPCs) as well as LLPCs in the bone marrow are likely to contribute to serum antibody levels, it was of interest to determine whether the mBC ELISPOT was measuring an independent attribute of the adaptive immune response in comparison to ELISA. Data were pooled following vaccination for all vaccinees in the Phase I/IIa trials. A significant positive correlation between IgG^+^ mBC-derived ASC and serum IgG antibodies was observed in volunteers for both MSP1_19_ (*r*_s_ = 0·50, *P *=* *0·011, *n *=* *25; Fig. 5a) and AMA1 (*r*_s_ = 0·49, *P *=* *0·010, *n *=* *26; Fig. 5d). However, following CHMI in the Phase IIa trial, analysis of the dC+35 responses showed that this observed relationship for MSP1_19_ (*n *=* *20; Fig. 5b) was not as strongly maintained, with similar results at dC+90 (Fig. 5c). For AMA1 (*n *=* *18) the relationship was marginally maintained at dC+35 (Fig. 5e) but lost significance by dC+90 (Fig. 5f). Overall these data suggest that malaria exposure affects serum IgG levels and/or the circulating mBC populations leading to a more complex relationship between the two than that observed following vaccination only.

### Migratory ASC can be detected 7 days after MVA booster vaccination but were not observed at selected time-points during and post-CHMI

**Figure 6:**
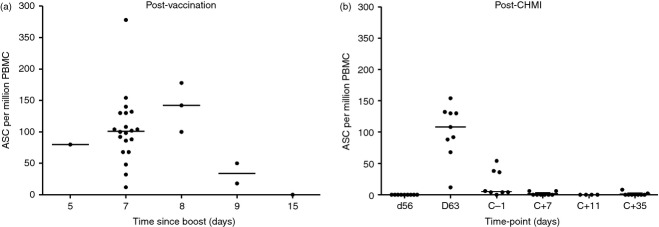
Measurement of 19 kDa C-terminus of merozoite surface protein 1 (MSP1_19_)-specific peripheral antigen-secreting cells (ASC) after vaccination and controlled human malaria infection (CHMI). (a) 19 kDa C-terminus of merozoite surface protein 1 (MSP1_19_)-specific ASC responses (per million peripheral blood mononuclear cells (PBMC) were assessed by *ex vivo* ELISPOT at the stated time-points after MVA-MSP1 booster vaccination. Volunteers all received MVA-MSP1 booster vaccination either alone (*n *=* *9) or co-administered with another MVA (*n *=* *18). The responses are reported as the exact number of days following booster vaccination. (b) MSP1_19_-specific ASC were also measured before (dC−1) and after CHMI at dC+7, dC+11 and dC+35. All volunteers (*n *=* *9) received MVA-MSP1 booster vaccination only and were assayed exactly 7 days later for the d63 time-point [shown as day 7 in (a)]. Eight out of nine volunteers underwent CHMI of which 4/8 progressed to dC+11 before diagnosis. Data are presented as dot plots showing individual and median responses.

Having shown that parasite exposure could perturb the relationship between serum IgG and peripheral mBC, as well as boost vaccine-induced MSP1_19_-specific mBC responses and lead to their induction in control volunteers, we sought to assess whether ASC responses themselves could be detected in peripheral blood. These responses can be typically observed in peripheral blood 7 days after a booster vaccination.^36^–^38^ We first performed *ex-vivo* ASC ELISPOT assays on all MSP1-immunized volunteers in the Phase IIa clinical trial, aiming to assay at the d7 time-point post-booster vaccination. The ELISPOT data for each volunteer are plotted according to the exact number of days assayed following booster vaccination (given this varied for reasons such as safety monitoring, and logistical limitations relating to clinic attendance and co-coordinating vaccinations with subsequent CHMI). In agreement with studies using different types of vaccines,^36^–^38^ MSP1_19_-specific ASC responses appeared to peak in the periphery around day 7–8 following a booster vaccination with MVA MSP1 (given either alone or co-administered;^30^ Fig. 6a), with the median response approximately 100 ASC/million PBMC. Lower responses were observed up to 2 days either side.

We subsequently performed the same assessment in nine volunteers vaccinated with MSP1 alone at all available time-points post-CHMI (Fig. 6b). The ASC responses in all nine volunteers were measured 7 days after booster vaccination (d63 in Fig. 6b equivalent to day 7 in Fig. 6a). Responses were next measured at 1 day before CHMI (dC−1/d72). At this time-point most volunteers showed no detectable MSP1_19_-specific ASC, although three volunteers still had residual responses around 40 ASC/million PBMC. Following CHMI, *ex vivo* ASC ELISPOT assays were performed at days C+7, +11 and +35, but responses at all time-points were considered negative for detectable ASC after subtracting background. In CHMI control volunteers receiving no previous vaccines, ASC responses were also negative at these time-points (data not shown). Hence, despite the noted boosting of MSP1_19_-specific mBC responses, we were unable to detect a migratory ASC population during infection at the limited number of time-points for which PBMC were available. As with mBC ELISPOT, DT controls were run alongside the vaccine antigen. No DT-specific ASC were detected at any time-point in the study, suggesting no bystander responses or LLPC displacement from the bone marrow (data not shown).

### CXCR3^+^ B cells begin to migrate away from the peripheral blood at the point of diagnosis

We next sought to assess the effect(s) of CHMI on the phenotype of peripheral B cells. Given that we had seen a decrease in peripheral blood mBC responses during CHMI (Fig. 3a,d), PBMC at selected time-points from six individuals vaccinated with MSP1 from the Phase IIa trial were stained with B-cell phenotypic markers as described in the Materials and methods and gated as shown in Fig. S2. Within the CD19^+^ population the subsets of B cells characterized through this staining strategy remained stable over time (Fig. S3). At the DoD a slight increase in classical, immature and atypical subsets (Fig. S3a,e,f) was observed in contrast to a slight decrease in naive B cells (Fig. S3b) but none of these changes reached significance. Plasma cells were seen to be increased in some volunteers at d63 but not during CHMI (Fig. S3d), in agreement with the ASC data (Fig. 6b), although it is possible that the sensitivity of detection may have been reduced because of the use of frozen cells for flow cytometry analysis in comparison to fresh PBMC used for the *ex vivo* ELISPOT.^39^

**Figure 7:**
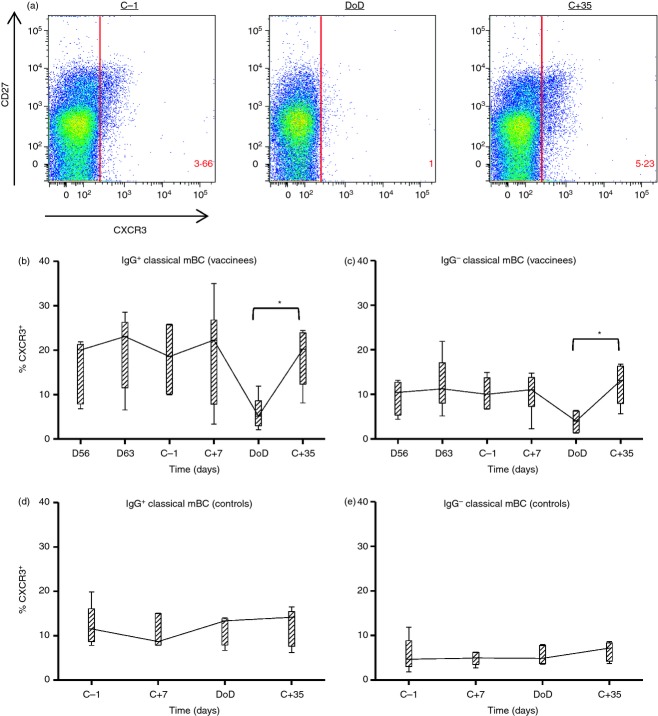
CXCR3^+^ memory B cells (mBC) in peripheral blood during blood-stage infection. Flow cytometry was used to identify CXCR3^+^ B cells before, during and after controlled human malaria infection (CHMI). (a) CXCR3 expression on ‘mature’ B cells, gated as lymphocytes, singlets, CD19^+^ and CD10^−^. Representative flow plots are shown for the dC−1, day of diagnosis (DoD) and dC+35 time-points. (b) % of IgG^+^ classical mBC expressing CXCR3 and (c) % of IgG^−^ classical mBC expressing CXCR3 in six volunteers vaccinated with merozoite surface protein 1 (MSP1). (d) % of IgG^+^ classical mBC expressing CXCR3 and (e) % of IgG^−^ classical mBC expressing CXCR3 in five control volunteers. Box and whisker plots show the median, range and interquartile range. Differences between time-points were analysed by Wilcoxon matched-pairs signed rank test (solid lines), *P <* *0·05.

Given there was no generalized loss of peripheral mBC populations at DoD, we subsequently assessed the CXCR3^+^ populations within each subset. CXCR3 is a marker associated with migration to areas of inflammation via the ligand CXCL9 (MIG).^40^ This marker is commonly expressed on mature B cells (CD19^+^, CD10^−^), particularly those that express CD27 and are class switched.^41^ In agreement with this, the majority of CXCR3^+^ CD19^+^ CD10^−^ B cells were found to be CD27^+^ at the dC−1 time-point (Fig. 7a). Interestingly, the total number of CXCR3^+^ CD19^+^ CD10^−^ B cells was observed to decrease at DoD in comparison to dC−1, and then recover by dC+35 (Fig. 7a). CXCR3^+^ cells comprised approximately 20% of the IgG^+^ classical mBC population at most time-points measured; however, this dropped to a median level of 5·2% at DoD (Fig. 7b). A significant difference was observed between the % CXCR3^+^ cells at DoD and dC+35 (*P *=* *0·0313), whereas the same trend was observed between dC−1 and DoD (*P *=* *0·065). Similarly, CXCR3^+^ IgG^−^ cells (which comprise approximately 10% of the classical mBC population at most time-points) decreased at DoD (median 4%) again significantly from dC+35 (*P *=* *0·0313; Fig. 7c). Similar observations to that observed with IgG^+^ classical mBC were also apparent for the activated mBC (see Supporting information, Fig. S4a) and atypical mBC (Fig. S4b) populations. In contrast to the MSP1-vaccinated volunteers, in unvaccinated infectivity control volunteers the % of cells expressing CXCR3 was stable over time for both IgG^+/−^ classical mBC (Fig. 7d,e) and all other B-cell subsets (Fig. S4d–f).

**Figure 8:**
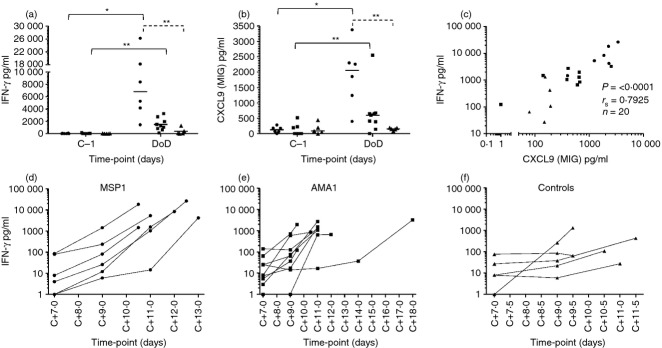
Serum levels of interferon-*γ* (IFN-*γ*) and CXCL9 before and during controlled human malaria infection (CHMI). Serum levels of (a) IFN-*γ* and (b) CXCL9 were assessed by ELISA in volunteers that received merozoite surface protein 1 (MSP1) vaccination (circles), apical membrane antigen 1 (AMA1) vaccination (squares) and unvaccinated control volunteers (triangles) before CHMI and at day of diagnosis (DoD). Individual responses and medians are shown. (c) Correlation between IFN-*γ* and CXCL9. The individual serum IFN-*γ* levels following CHMI and during blood-stage infection are shown for (d) MSP1 vaccinees, (e) AMA1 vaccinees and (f) unvaccinated controls. Differences between time-points were analysed by Wilcoxon matched-pairs signed rank test (solid lines); differences between vaccinated volunteers and CHMI controls were analysed by Mann–Whitney test (dashed lines); *P <* *0·05 and **P <* *0·01.

Following the observation that fewer CXCR3^+^ B cells were observed in the peripheral blood of vaccinees at DoD, we next investigated up-regulation of the inflammatory serum cytokines IFN-*γ* and CXCL9 that may be driving migration. Serum ELISA showed significant up-regulation of IFN-*γ* and CXCL9 in both MSP1-(*P *=* *0·0313 for both) and AMA1-(*P *=* *0·0078 for both) vaccinated volunteers but not in controls at DoD compared with dC−1 (Fig. 8a,b). At DoD (but not dC−1) there was also a significant difference in the serum levels of both IFN-*γ* and CXCL9 between MSP1-vaccinated volunteers and control volunteers. The IFN-*γ* and CXCL9 responses by ELISA strongly correlated with one another (Fig. 8c). An increase in serum IFN-*γ* was observed during the period of blood-stage infection (before diagnosis) in individuals vaccinated with MSP1 (Fig. 8d) and AMA1 (Fig. 8e), but to a lesser extent in control volunteers (Fig. 8f). Overall, these higher levels of IFN-*γ* and CXCL9 could account for an increased level of CXCR3^+^ B-cell migration out of the periphery in MSP1 vaccinees.

## Discussion

Unlike many human pathogens that induce high levels of protective immunological memory after a single infection,^42^ the acquisition of NAI against *P. falciparum* malaria requires repeated exposure over significant periods of time.^1^ Such NAI is largely antibody-mediated against the blood-stage parasite forms, but is non-sterilizing – leading to asymptomatic carriage of parasites in older individuals living in endemic areas. Although many potential hypotheses could be proposed as to why such immunity arises, in recent years a number of rodent malaria and immuno-epidemiological studies have argued that the inefficient acquisition of protective antibodies is linked to parasite-related dysregulation of B-cell function.^23^ An alternative approach to address these important questions is provided by access to established CHMI models;^43^,^44^ however, to date these highly valuable opportunities have not been used to assess the impact of experimental infection on human B cells. Here we report for the first time on the impact of CHMI on B cells in malaria-naive individuals as well as those previously vaccinated against two candidate merozoite antigens.

Initially we assessed mBC induction in healthy adult volunteers immunized with ChAd63-MVA virus-vectored vaccines encoding the blood-stage merozoite antigens MSP1 and AMA1. This highly immunogenic subunit vaccine delivery platform has undergone recent clinical development for a range of difficult infectious disease targets,^45^,^46^ and these vaccine candidates were previously reported to induce high levels of antigen-specific T cells as well as substantial serum IgG antibody responses in healthy adult UK volunteers.^28^,^29^ Here, we showed that antigen-specific mBC responses were primed and boosted against both antigens following ChAd63-MVA MSP1 or AMA1 vaccination. The levels of antigen-specific mBC were higher than those reported in healthy US adults following three immunizations of MSP1 or AMA1 protein vaccine formulated with the aluminium-based adjuvant Alhydrogel, although slightly lower than when the Toll-like receptor 9 agonist CPG7909 was also included in the formulation.^25^ In contrast, the same Alhydrogel-CPG7909 adjuvant failed to show improved mBC responses over Alhydrogel alone in a Malian adult cohort.^47^ It remains to be seen how virus-vectored vaccines would perform for mBC induction in an endemic population, although they appear to generate comparable T-cell immunogenicity in endemic adults.^48^

We also recently completed a series of Phase IIa studies aimed at assessing vaccine efficacy. Overall, all vaccinated volunteers (bar one co-immunized with MSP1 and AMA1) developed blood-stage infection, and there was no measurable impact of the vaccines on blood-stage parasite multiplication rates.^30^ However, these studies provided a unique opportunity to assess the possible effects of CHMI on B cells in both vaccinated individuals as well as non-immunized infectivity control volunteers. Cohorts of controls were assessed from three separate studies where infection was initiated by the bites of five infected *Anopheles stephensi* mosquitoes harbouring 3D7 clone sporozoites. Following liver-stage infection, merozoites rupture out of infected hepatocytes and initiate the blood-stage infection. The peak of the first wave of parasitaemia is typically detected by sensitive quantitative PCR monitoring on day 7, and on average blood-stage infection is detected by thick-film microscopy on days 10–11 in controls (typically at quantitative PCR levels of ˜ 10 000 parasites/ml blood), at which point drug treatment is initiated to clear the infection.^30^,^35^ Here we observed that 14/17 malaria-naive control volunteers subjected to CHMI developed mBC and serum IgG responses to MSP1_19_ after a single controlled infection, in contrast to AMA1 where responses were largely undetectable. The MSP1_19_-specific mBC response appeared comparable, if not stronger, than that observed following ChAd63-MVA MSP1 vaccination alone. These immune responses positively correlated with each other, and also the duration of blood-stage parasite exposure. In contrast, responses to both antigens have been reported in the field,^10^,^12^,^13^,^15^,^16^,^49^ but in agreement with our data, results from low transmission areas have shown MSP1_19_ to generate more robust antibody responses than AMA1, as well as mBC responses after a single reported infection.^14^ We also assessed mBC induction following co-administration of the ChAd63-MVA vaccines encoding MSP1 and AMA1. In this case, responses to both antigens were on average lower in comparison to single vaccine administration; however, these data confirm that it is possible to mount responses against both antigens at the same time. These data suggest that in the context of primary acute human *P. falciparum* infection, antibody and mBC induction occur more consistently and effectively for MSP1_19_ than AMA1. Moreover, *in vitro* functional assays measuring the ability of purified IgG to neutralize red blood cell invasion by merozoites have shown anti-AMA1 IgG to be quantitatively more effective than anti-MSP1.^50^ It therefore seems possible that the parasite may have evolved to evoke primary immune responses against less susceptible antigenic targets – a possible reflection of exposure time^51^ or antigen abundance within the blood-stage merozoite. Alternatively, such responses may be a simple consequence of immuno-dominance hierarchies that inevitably arise when the immune system is exposed to so many antigens in the context of a highly complex pathogen. Although not tested here, repeated or prolonged exposure may therefore be required before responses develop to AMA1, as noted in the field where the mBC repertoire expands with increased exposure.^15^

In the case of immunized volunteers, mBC responses for both MSP1_19_ and AMA1 were clearly boosted following CHMI and drug clearance. In agreement with the above data suggesting a more substantial immunogenicity for MSP1_19_ than AMA1 in the context of parasite infection, the resting mBC level approximately 3 months post-drug treatment was significantly higher only for MSP1-vaccinated volunteers in comparison to vaccination only responses at the most similar time-point. A ‘division in labour’ of the mBC response can also be seen in natural infection whereby responses increase in antigen breadth, but not overall magnitude,^15^ which would agree with the AMA1 data here, although in this study we did not assess *de novo* responses against other antigens in these vaccinees. A field study in Mali, a high transmission area, has shown similar boosting of MSP1-specific and AMA1-specific mBC responses following an acute episode, with only a marginal increase in long-term mBC resting levels^10^ – again very similar to the data shown here following a single CHMI. Studies in mice have also shown that MSP1_19_-vaccine-induced mBC are re-stimulated to produce ASC and antibodies following *Plasmodium berghei* parasite challenge.^52^ Importantly, and in contrast to data from the *P. yoelii* mouse model,^22^ these data confirm that vaccine-induced mBC responses are neither reduced nor ablated following a single exposure to blood-stage malaria parasites. Although we have only been able to assess after a single CHMI, these data are encouraging for malaria vaccine development and provide an impetus for similar studies in the future to address the impact of natural and repeated malaria exposure on vaccine-induced mBC.

In agreement with a large number of other human vaccine studies,^25^,^53^,^54^ we also observed that antigen-specific IgG serum antibody titres correlated with the mBC response post-vaccination. Such IgG titres are probably due to antibody production from SLPCs and/or LLPCs; however, these data indicate that these plasma cell populations are probably proportional to the induced mBC response. In contrast, this relationship was less well maintained after CHMI, in agreement with some studies of natural exposure,^13^,^15^ but not others.^16^ It is possible that during infection, serum IgG may have been depleted through binding to parasite antigen, and mBC will have probably proliferated into new SLPCs or LLPCs. Two field studies have also noted possible polyclonal activation of DT and tetanus toxoid-specific bystander mBC responses following episodes of clinical malaria.^10^,^15^ In contrast, tetanus toxoid-specific mBC responses were reported to be stable following asymptomatic or persistent infection of Kenyan children.^12^ We also observed no obvious detrimental impact of either vaccination or malaria exposure on bystander mBC responses to DT. We also noted no apparent increase in the ‘atypical mBC’ subset following a single CHMI. This is not surprising, given that field data on this subset suggest that the expansion of this population associates with levels of increasing or chronic exposure.^10^,^20^,^21^,^55^ More recent data have suggested that these cells produce functional IgG and may arise from constant activation, rather than impaired memory function.^56^ It is possible that CHMI studies in endemic countries may be more suited to assessing the impact of controlled infection on these cell populations.

A peak of migratory antigen-specific IgG^+^ ASC was detected in the peripheral blood 7–8 days following the MVA booster vaccination. These observations are typical of an antigen-specific secondary immune response^57^ with similar observations in humans seen with seasonal influenza vaccination,^58^ pneumococcal conjugate vaccines^59^ and tetanus toxoid.^60^ These ASC populations observed at days 7–8 in the peripheral blood are referred to as plasmablasts, and probably reflect cells that have arisen from mBC re-stimulation in the lymphoid system, and now en route to their niche within the bone marrow where they will remain as plasma cells. However, following CHMI, we were unable to detect MSP1_19_-specific plasmablast responses, although PBMC samples were available from a limited number of time-points, and none between drug treatment and 35 days post-challenge. Expanded total plasmablast populations have been seen after infection in the field,^14^ and in the *Plasmodium chabaudi* mouse model, MSP1_19_-specific ASC were also observed in the blood 10–25 days following a primary infection.^61^ A more detailed kinetic analysis of ASC responses is required in future CHMI studies to assess this in more detail.

During these studies, we also noted a significant decrease in the number of MSP1_19_-specific mBC in the peripheral blood at the time-point of diagnosis. Decreases in absolute numbers of peripheral lymphocytes have been previously reported following CHMI, although % CD19^+^ cells within the lymphocyte population remained stable.^62^ Interestingly, the classical mBC population, within which the MSP1_19_-specific population would be located, remained stable during this period. Given that no significant reduction in AMA1-specific or bystander DT-specific mBC was observed, we concluded that a generalized migration or loss of peripheral blood mBC is unlikely to account for the reduced levels of MSP1_19_-specific mBC.

One possible explanation is that significant numbers of MSP1_19_-specific mBC are being sequestered away from the periphery as the blood-stage infection progresses, most likely in secondary lymphoid organs, where they are retained through MSP1_19_ antigen presentation. The fact that a significant decrease was observed during CHMI for MSP1 (and not AMA1) is in agreement with the above data regarding MSP1 immuno-dominance in the context of natural infection. To further investigate this hypothesis, we assessed the expression of the migratory chemokine receptor marker CXCR3. CXCL9 (MIG) is induced by IFN-*γ* and plays an important role in the recruitment of CXCR3^+^ cells to sites of inflammation.^63^,^64^ The up-regulation of CXCL9 by antigen-specific T cells has been previously demonstrated^65^ and CXCL9 and IFN-*γ* have been shown to correlate in another virus-vectored malaria vaccine regimen.^66^ Our original ChAd63-MVA vaccine candidates for MSP1 and AMA1 were developed to assess the possibility that strong cellular immunity, in conjunction with antibodies, may better impact on blood-stage parasite growth than antibodies alone.^28^,^29^ Although the Phase IIa efficacy trial showed this not to be the case,^30^ we confirmed here that significant levels of serum IFN-*γ* were observed during blood-stage infection in vaccinees, in contrast to the unvaccinated controls, with previous MSP1 vaccination again leading to higher levels than AMA1. In agreement with this observation immune recognition of blood-stage antigens has been reported in a blood-stage challenge model, accompanied by a reduction in peripheral blood lymphocytes.^67^ In contrast, control volunteers lacking pre-existing malaria-specific T cells showed lower levels of serum IFN-*γ*, similar to those previously reported in other studies,^68^,^69^ and therefore less marked migration of CXCR3^+^ B cells away from the periphery. An alternative possibility is that by the time of diagnosis there is induction of apoptosis in activated MSP1_19_-specific CXCR3^+^ B cells. However, given that these malaria-specific responses are significantly boosted following CHMI and that B-cell populations appeared stable in controls, it seemed unlikely that high levels of cell death would have led to the observed decrease in MSP1_19_-specific mBC at these levels of parasitaemia.

In summary, NAI to blood-stage malaria arises over multiple exposures and there remains a need to better understand whether this is due to dysregulated B-cell responses. Here we have investigated this for the first time in the CHMI model. Although our data compare favourably with those from low transmission areas, it remains impossible to study higher levels of parasitaemia in the CHMI model comparable to those observed in high transmission areas. Nevertheless, our data remain encouraging for vaccines and suggest that vaccine-induced mBC responses can be effectively boosted by low-level blood-stage parasite exposure, with no evidence of B-cell ablation of malaria-specific or bystander responses.

## Supporting Information

Figure S1 Phase I/IIa vaccine trial timelines.

Figure S2 B-cell gating strategy.

Figure S3 B-cell subset composition of total B-cell pool over time.

Figure S4 CXCR3 expression on B-cell subsets.
