# Supplementary figures and images for: Analysis of human B-cell responses following ChAd63-MVA MSP1 and AMA1 immunization and controlled malaria infection
Source: Immunology. 2014 Mar 11;141(4):628–44. doi: 10.1111/imm.12226 (PMC3956436; doi:10.1111/imm.12226)

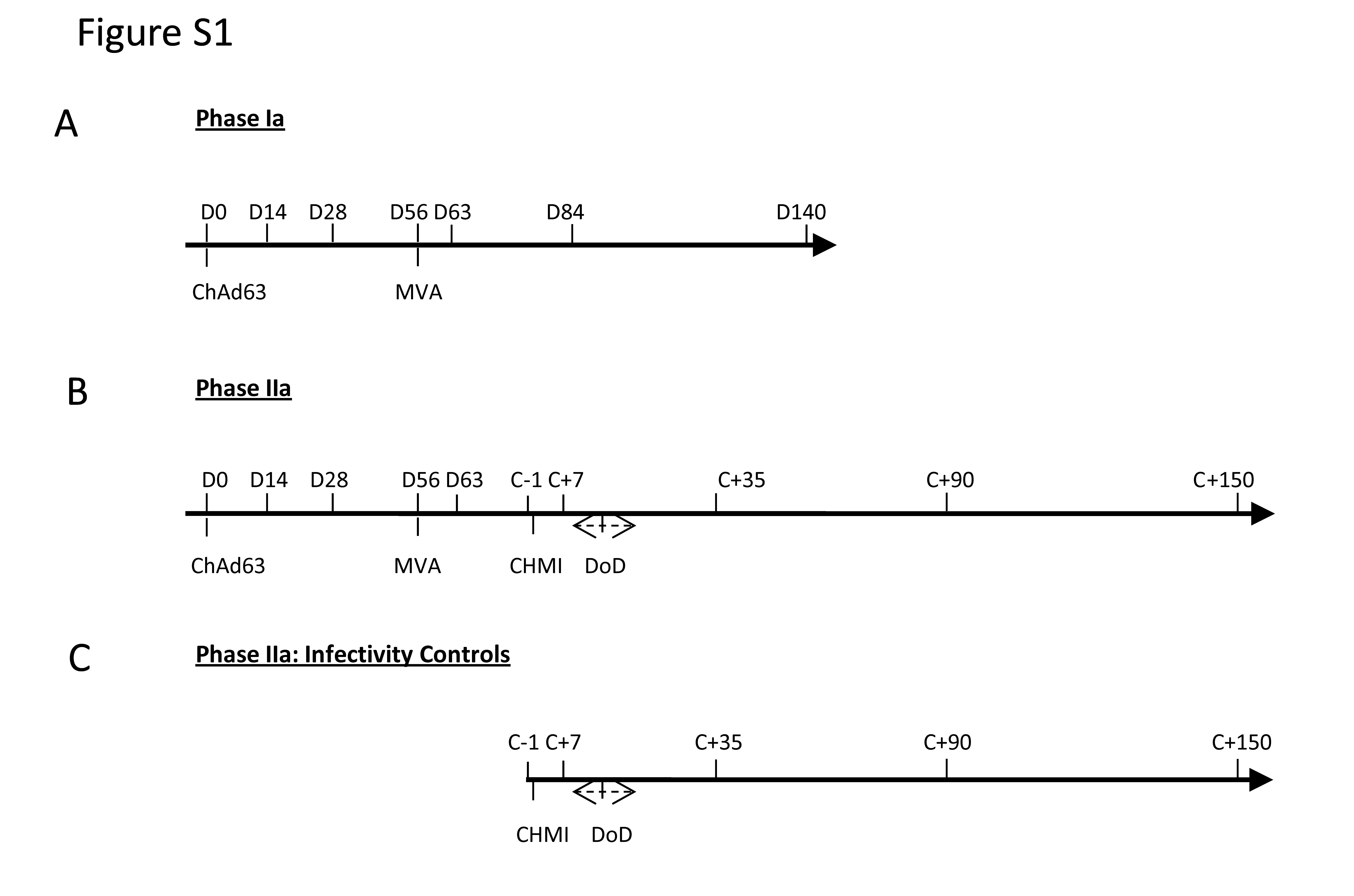

Supplement: Figure S1 — Phase I/IIa vaccine trial timelines. [file imm0141-0628-sd1.tif]

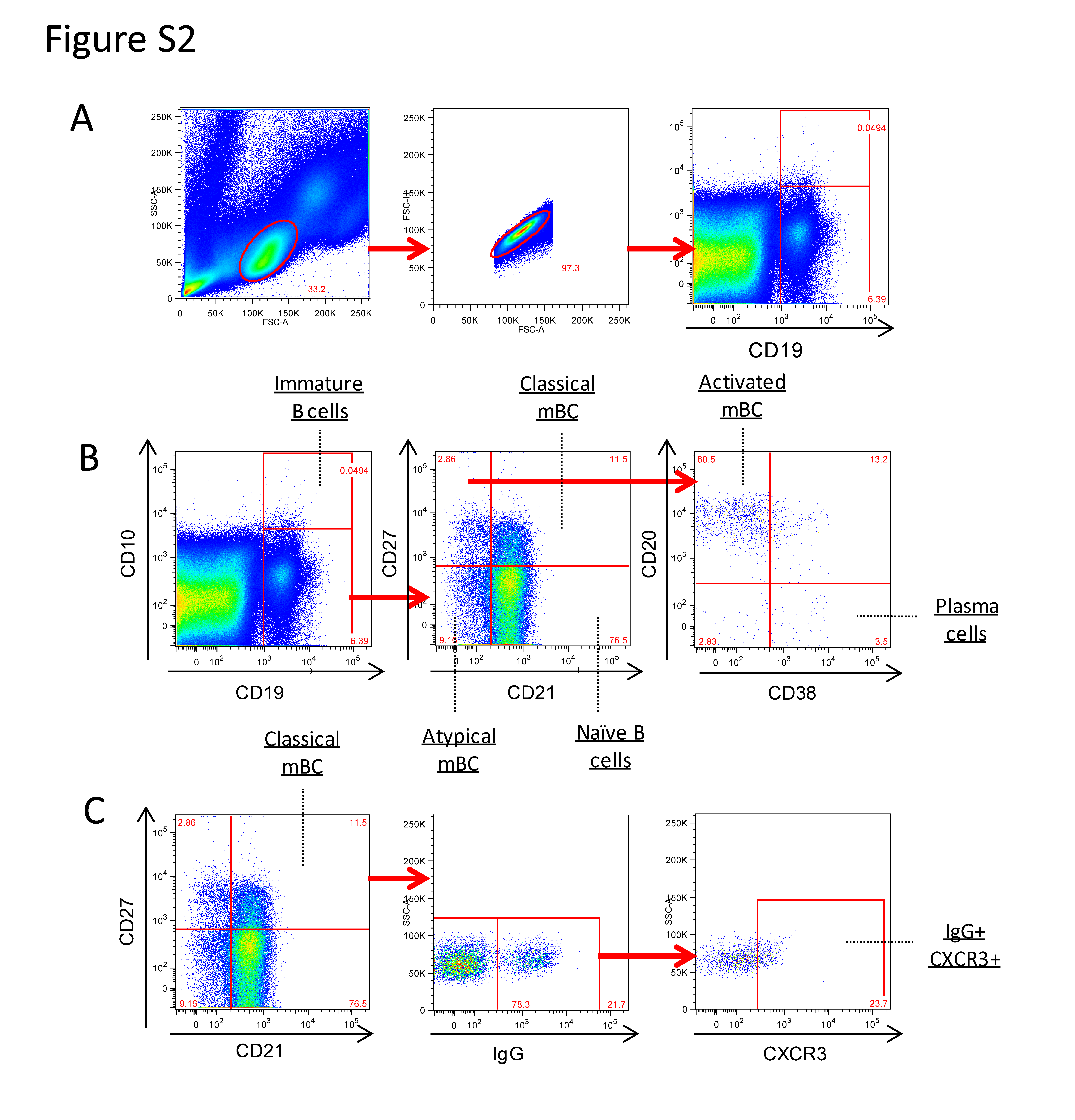

Supplement: Figure S2 — B-cell gating strategy. [file imm0141-0628-sd2.tif]
