# Supplementary material for: Analysis of human B-cell responses following ChAd63-MVA MSP1 and AMA1 immunization and controlled malaria infection
Source: Immunology. 2014 Mar 11;141(4):628–44. doi: 10.1111/imm.12226 (PMC3956436; doi:10.1111/imm.12226)
Supplement: Supplementary file 5 [file imm0141-0628-sd5.docx]

## Figure S1: Phase I/IIa vaccine trial timelines.

mBC responses from key time-points within Phase Ia ([1](#_ENREF_1), [2](#_ENREF_2)) and Phase IIa ([3](#_ENREF_3)) clinical trials were analysed in this study. **(A)** In Phase Ia studies, ChAd63 priming vaccination on day 0 was followed 8 weeks later with MVA booster vaccination. **(B)** In Phase IIa studies, controlled human malaria infection (CHMI) followed MVA booster vaccination on average 17 days later (range 14-23). **(C)** Phase IIa infectivity control volunteers underwent CHMI without previous vaccination in parallel with Phase IIa vaccinated volunteers. Time-point dC-1 indicates the day before CHMI. Thereafter, time-points are referred to as the number of days post-infection, e.g. dC+35 means 35 days after CHMI. Non-protected and control volunteers were diagnosed with microscope-patent blood-stage malaria infection on average 10-11 days after challenge at which point they received anti-malarial drug treatment ([3](#_ENREF_3)). The time window for typical day of diagnosis (DoD) is indicated. Prior to this, blood sampling occurred according to trial protocol during the period of blood-stage infection (from dC+7 until DoD). The next follow-up time-point was dC+35.

## Figure S2: B cell gating strategy.

**(A)** Whole PBMC were gated first by lymphocytes (left) and then singlets (centre) before gating on CD19^+^ to identify B cells (right). **(B)** Immature B cells were gated as CD19^+^ CD10^+^ (left), whilst CD19^+^ CD10^–^ cells were further gated using CD21 and CD27 (centre) to distinguish naïve B cells, classical mBCs and atypical mBCs. The CD21^–^ CD27^+^ population was further gated using CD20 and CD38 (right) to distinguish activated mBC and plasma cells. **(C)** Classical mBC populations were gated using IgG^+/–^ (centre) and then CXCR3 (right) to identify further subsets of interest.

## Figure S3. B cell subset composition of total B cell pool over time.

Flow cytometry was used to identify relative changes to B cell subsets over time in 6 volunteers receiving the MSP1 vaccine regime followed by CHMI. Subsets are expressed as a % of total B cells, defined as number of gated events for each subset out of total CD19^+^ events (after subtraction of undefined subsets as described in Methods). **(A)** classical mBC, **(B)** naïve B cells, **(C)** activated mBC, **(D)** plasma cells, **(E)** immature B cells, and **(F)** atypical mBC. Differences between time-points identified in the Results were analysed by Wilcoxon matched-pairs signed rank test.

## Figure S4: CXCR3 expression on B cell subsets.

Flow cytometry was used to identify CXCR3^+^ B cells before, during and after CHMI. **(A)** % of activated mBC expressing CXCR3, **(B)** % of atypical mBC expressing CXCR3 and **(C)** % of naïve B cells expressing CXCR3 in 6 volunteers vaccinated with MSP1 prior to CHMI. **(D)** % of activated mBC expressing CXCR3, **(E)** % of atypical mBC expressing CXCR3 and **(F)** % of naïve B cells expressing CXCR3 in 5 unvaccinated infectivity control volunteers. Differences between time-points were analysed by Wilcoxon matched-pairs signed rank test (solid lines), **P*<0.05.

## Supplementary References

1. Sheehy, S. H., C. J. Duncan, S. C. Elias, S. Biswas, K. A. Collins, G. A. O'Hara, F. D. Halstead, K. J. Ewer, T. Mahungu, A. J. Spencer, K. Miura, I. D. Poulton, M. D. Dicks, N. J. Edwards, E. Berrie, S. Moyle, S. Colloca, R. Cortese, K. Gantlett, C. A. Long, A. M. Lawrie, S. C. Gilbert, T. Doherty, A. Nicosia, A. V. Hill, and S. J. Draper. 2012. Phase Ia Clinical Evaluation of the Safety and Immunogenicity of the Plasmodium falciparum Blood-Stage Antigen AMA1 in ChAd63 and MVA Vaccine Vectors. *PLoS One* 7:e31208.

2. Sheehy, S. H., C. J. Duncan, S. C. Elias, K. A. Collins, K. J. Ewer, A. J. Spencer, A. R. Williams, F. D. Halstead, S. E. Moretz, K. Miura, C. Epp, M. D. Dicks, I. D. Poulton, A. M. Lawrie, E. Berrie, S. Moyle, C. A. Long, S. Colloca, R. Cortese, S. C. Gilbert, A. Nicosia, A. V. Hill, and S. J. Draper. 2011. Phase Ia Clinical Evaluation of the Plasmodium falciparum Blood-stage Antigen MSP1 in ChAd63 and MVA Vaccine Vectors. *Mol Ther* 19:2269-2276.

3. Sheehy, S. H., C. J. Duncan, S. C. Elias, P. Choudhary, S. Biswas, F. D. Halstead, K. A. Collins, N. J. Edwards, A. D. Douglas, N. A. Anagnostou, K. J. Ewer, T. Havelock, T. Mahungu, C. M. Bliss, K. Miura, I. D. Poulton, P. J. Lillie, R. D. Antrobus, E. Berrie, S. Moyle, K. Gantlett, S. Colloca, R. Cortese, C. A. Long, R. E. Sinden, S. C. Gilbert, A. M. Lawrie, T. Doherty, S. N. Faust, A. Nicosia, A. V. S. Hill, and S. J. Draper. 2012. ChAd63-MVA–vectored Blood-stage Malaria Vaccines Targeting MSP1 and AMA1: Assessment of Efficacy Against Mosquito Bite Challenge in Humans. *Mol Ther* 20:2355-2368.
